# Convenient Reparation of SiC-Coated C/C Composites by the Slurry Painting Method

**DOI:** 10.3390/ma17184515

**Published:** 2024-09-14

**Authors:** Hui Peng, Xiaohong Shi, Fan Jiao, Xutong Ti, Linyi Du

**Affiliations:** 1 State Key Laboratory of Solidification Processing, Shaanxi Key Laboratory of Fiber Reinforced Light Weight Composites, Northwestern Polytechnical University, Xi’an 710072, China; ph15256523687@163.com (H.P.); jiaofan1021@163.com (F.J.); txt1996@mail.nwpu.edu.cn (X.T.); d2910286136@163.com (L.D.); 2 Henan Key Laboratory of High Performance Carbon Fiber Reinforced Composites, Carbon Matrix Composites Research Institute, Henan Academy of Sciences, Zhengzhou 450046, China

**Keywords:** repair coating, C/C composites, oxidation, slurry painting

## Abstract

SiC-coated C/C composites with mechanical damage were repaired by the heat treatment method and slurry painting–preoxidation. The effects of different process parameters on the microstructure, interface bonding and oxidation resistance of Si-SiC repair coatings at 1773 K for 10 h were studied. The results show that the repair coating is tightly bonded to the original coating and the C/C substrate, and there is no obvious interface. Under the optimal parameters, the weight reduction in the repaired specimen merely amounted to 0.32% subsequent to oxidation at 1773 K for 10 h, and the mass loss was 74.79% lower than that of the damaged specimen, being proximate to that of the intact specimen. The objective of this work lies in achieving a greater density of the coating within the repair zone by manipulating the diverse powder ratios and preoxidation temperatures in the repair slurry, thereby safeguarding the C/C composite material against oxidation during its service. It offers a convenient and highly efficient approach for the repair of coatings with substantial size defects, significantly prolonging the service life of the material.

## 1. Introduction

A carbon/carbon (C/C) composite is an advanced composite material based on a carbon fiber-reinforced carbon matrix. With its low density, low thermal expansion coefficient, high thermal conductivity and high temperature mechanical properties, it is often used as a thermal protection component of aircraft. It is an important candidate material in aerospace and other high-tech fields [1,2,3,4]. Studies have shown that the material begins to oxidize in an aerobic environment above 370 °C [5]. When the oxidation weight loss reaches 1%, the strength decreases by 10%. When the oxidation weight loss reaches 10%, the strength decreases by 50% [6,7], which seriously limits the wide application of C/C composite components [8].

An effective way to solve this problem is to prepare an antioxidant ceramic coating on the surface of C/C composites, which can improve the oxidation resistance of C/C composites by completely isolating them from the aerobic environment. Because SiC ceramics have good physical and chemical compatibility with C/C composites [9], they can greatly reduce the interfacial thermal stress between the coating and the substrate, so they are often used as anti-oxidation coating materials for C/C composites [10,11,12]. In addition, the oxide product of SiC is SiO_2_ in the glass phase [13,14,15]. On the one hand, it can fill the cracks, holes and other defects generated during the service process, and on the other hand, it can further prevent the diffusion of oxygen to the C/C composite matrix [16,17,18].

However, the SiC coating of C/C composites is often prone to surface coating damage during transportation and service [19]. If the damaged coating is not treated in time, it will lead to the collapse of the entire coating system, resulting in serious consequences [20]. At present, the commonly used method is to replace the entire coating, which means that it will greatly increase the cost of use and waste raw materials to a large extent. Compared with the method of directly replacing the entire coating, a more economical and effective method is to repair the damaged area of the coating. Using this method can greatly extend the service life of the material and reduce the cost of use [4]. Si-SiC ceramics have good physical and chemical compatibility with the C/C composite matrix and can form a self-healing phase with low oxygen permeability under a high-temperature aerobic environment, making it a common material system for the thermal protection coatings of C/C composites [21,22,23,24].

**Figure 1:**
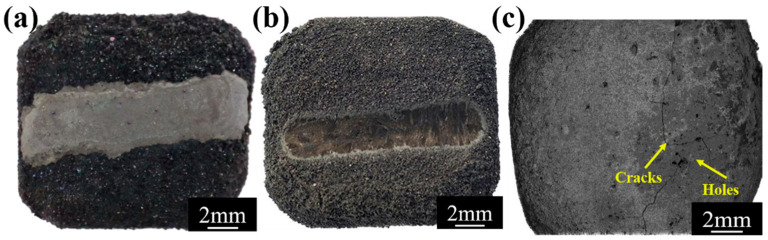
The macroscopic morphology of (**a**) the repaired sample, (**b**) the pretreated sample and (**c**) the damaged sample.

The slurry painting method entails dissolving a solvent with the powder of a pre-prepared coating [25,26,27], followed by adding a specific dispersant and binder to uniformly coat the coating material on the surface of the substrate in the form of slurry or dipping the C/C substrate in the slurry. After that, the coating material is adhered to the surface of the substrate, and then the coated coating is subjected to heat treatment in a special atmosphere to finally obtain the required coating. Due to the convenient operation of the slurry painting method and the simple and easy control of the process, it has gradually attracted attention in recent years. In this work, a Si-SiC ceramic was used as the repair powder, and the mechanically damaged SiC-coated C/C composites were repaired by heat treatment and the slurry painting–preoxidation method. There are macroscopic cracks, holes and other defects on the surface of the damaged sample, but the matrix is not damaged (as shown in Figure 1c). The effects of process parameters on the microstructure, interface and oxidation resistance of the repaired samples were studied.

## 2. Experiment

### 2.1. Preparation and Pretreatment

The carbon/carbon composite material with a density of about 1.6–1.7 g/cm^3^ was selected as the matrix, and the cutting machine was used to cut it into a cuboid of 15 × 10 × 5 mm^3^, and sandpaper with a roughness of 300 mesh was used to polish the corners and edges of the sample. We prevented the build-up of thermal stress concentration. Then, after guiding the angle, the samples were ultrasonically cleaned with anhydrous ethanol for 30 min and dried at 70 °C for 10–12 h. SiC coating was prepared on the surface of carbon/carbon composites by the pack cementation infiltration method [28,29,30]. Si (≥99.7 purity, Tianjin Fengyue Chemical Reagent Co., Ltd., Tianjin, China) (75–85 wt%), C (≥99.7 purity, Xi’an Graphite Plant, Xi’an, China) (10–20 wt%) and Al_2_O_3_ (≥99.7 purity, Sinopharm Chemical Reagent Co., Ltd., Shanghai, China) (5–15 wt%) powders were mixed evenly. The SiC-coated carbon/carbon composites were obtained by coating the carbon/carbon composites after chamfering them in the mixed powder and allowing them to react in an argon atmosphere at 2173 K–2373 K for 2–4 h (ZGS-350 high-temperature graphitization furnace). Then, the SiC-coated carbon/carbon composites were cleaned under the above conditions and dried in an oven at 80 °C for 10 h. In order to verify the feasibility of the repair scheme, the surface of SiC-coated carbon/carbon composites was sandblasted to simulate mechanical damage. The sandblasting medium was 800 μm Al_2_O_3_ sand, the sandblasting pressure was 0.8 MPa, and the sandblasting time was 15–20 s.

A U-shaped defect with a depth of 0.2–0.4 mm and a width of 2–4 mm was polished on the surface damage area of SiC-coated carbon/carbon composites using a file (as shown in Figure 1b), and the edge was polished with sandpaper. On the one hand, this can prevent the concentration of thermal stress from building up, and on the other hand, this can increase the contact area between the repair agent and the area to be repaired. Then, the pretreated SiC-coated carbon/carbon composites were cleaned and dried under the above conditions.

### 2.2. Repair of Damaged Coating

#### 2.2.1. Initial Repair by Heat Treatment Method

The slurry approach was employed to handle the prearranged powder. Si (75–85 wt%), C (10–20 wt%), and Al_2_O_3_ (5–15 wt%) powders were utilized as raw materials, with anhydrous ethanol serving as the solvent for the preparation of the slurry. On the one hand, the Si powder is capable of reacting with the C powder to form SiC; on the other hand, it can react with the C/C substrate, resulting in a closer combination of the coating and the substrate. The Al_2_O_3_ powder is able to reduce the sintering temperature of the powder and facilitate the formation of a denser ceramic structure. The above slurry was magnetically stirred for 6–10 h (HJ-1 magnetic stirrer), then painted in a U-shaped defect, and then the sample was dried in an oven at 80 °C for 10 h. In addition, Si (75–85 wt%), C (10–20 wt%) and Al_2_O_3_ (5–15 wt%) powders were mixed by ball milling for 20–24 h to obtain dry powders (SFM-1 planetary ball-mill). The coated sample was wrapped in the above ball-milled mixed powder and reacted in an argon atmosphere at 2173 K–2373 K for 2–4 h to obtain an initially repaired SiC-coated C/C composite. Finally, the initial repaired samples were washed under the above conditions and dried in an oven at 80 °C for 10 h. The first repair of the pretreated sample by the heat treatment method can provide a certain antioxidant basis for the slurry painting–preoxidation method and avoid the oxidation of the C/C matrix during the subsequent slurry coating–oxidation repair.

#### 2.2.2. Slurry Brushing–Preoxidation Method for Further Repair

The initial repaired samples were further repaired by the slurry painting–preoxidation method. Si (25–35 wt%), Al_2_O_3_ (2.5–3.5 wt%) and SiC (≥99.7 purity, Henan Fanrui Research Institute of Composite Material, Kaifeng, China) (61.5–72.5 wt%) powders were used as raw materials, and the silica sol solution (≥99.7 purity, Qingdao Haiwan Group Co., Ltd., Qingdao, China) with a mass percentage of 5–10% was used as a solvent to prepare the slurry. On the one hand, the Si powder within the raw material can serve as a bonding phase to bond the SiC powder, given that SiC does not undergo melting at high temperatures. On the other hand, Si can form a flowing SiO_2_ glass phase in an oxygen-rich environment at elevated temperatures. This can not only further fill the defects such as pores and cracks in the coating but also impel the solid materials during its flow process, thereby rendering the coating more dense. The above slurry was stirred by magnetic stirring for 6–10 h, then brushed on the pretreatment site of the initial repair sample, and then the sample was dried in an oven at 80 °C for 10 h. The obtained samples were placed in a high-temperature oxidation furnace and reacted at 1400–1500 °C for 30 min in an air atmosphere to obtain a completely repaired sample. On the one hand, the slurry brushing–preoxidation method can increase the thickness of the coating in the repair area and control the thickness of the coating in the repair area to be close to the thickness of the original coating. On the other hand, the SiO_2_ glass in the slurry solvent has extremely low oxygen permeability, which can effectively prevent oxygen from diffusing into the C/C matrix. And it has certain fluidity at high temperature, which can effectively heal defects such as cracks and holes generated during the preparation of the coating by the embedding infiltration method, making the coating denser and significantly improving the oxidation resistance of the coating (as shown in Figure 1a).

The raw materials utilized in the two repair procedures are disparate for the ensuing reasons. The principal function of the first repair raw material, which contains a considerable amount of Si powder, is to react with the C powder to form SiC. The second repair process features a relatively brief heat treatment duration; thus, a substantial quantity of SiC is directly incorporated. Moreover, since the second repair process occurs in an aerobic atmosphere, no C powder is added to the raw material to avert oxidation during the heat treatment process, thereby avoiding the formation of holes and other defects.

#### 2.2.3. Process of Repairing Damaged Coating

**Figure 2:**
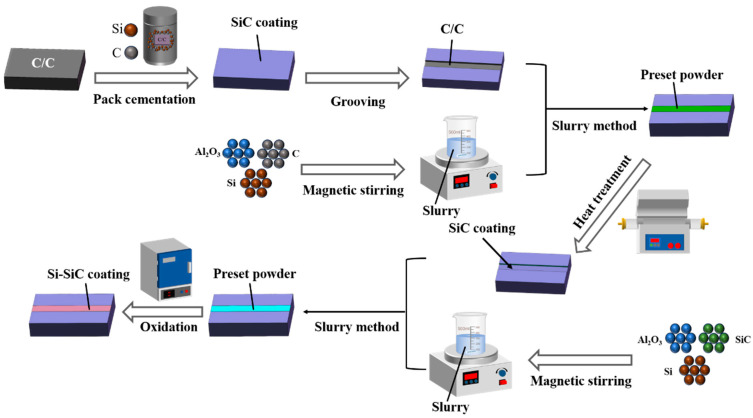
Repair schematic diagram of C/C composite surface damage coating.

The experimental process is shown in Figure 2 and the parameters of the oxidation process are shown in Table 1. In order to facilitate the distinction between the samples, the undamaged SiC-coated C/C composites are named undamaged samples and the unrepaired specimens are named damaged specimens. The repaired samples were named as Sample-1, Sample-2 and Sample-3, according to different process parameters.

### 2.3. Microstructure Characterization

The phase composition and crystalline state of the repaired samples before and after oxidation were analyzed by X-ray diffraction (XRD, X ‘pert PRO MPD, PANalytical B.V., Almelo, The Netherlands). The surface and cross-sectional morphology of the oxide layer before and after the repair sample was observed by field emission scanning electron microscopy (SEM, NovaTM Nano SEM450 and TM4000PLUS, FEI Company, Hillsboro, OR, USA and JEOL Ltd., Mitaka, Japan) equipped with an energy dispersive spectrometer (EDS, Oxford INCA, Oxford Instruments, Oxford, UK.).

### 2.4. Oxidation Test

The isothermal oxidation test at 1773 K in air was carried out in a high-temperature oxidation furnace (HGXSL industrial resistance furnace, Hefei Kejing Materials Technology Co., Ltd, Hefei, China) to evaluate the oxidation resistance of undamaged/damaged/repaired samples. The weight change (W%) is calculated according to Formula (1):

W% = (m_1_ − m_0_)/m_0_ × 100% (1)

where m_1_ is the weight after oxidation and m_0_ is the initial weight.

## 3. Results and Discussion

### 3.1. Characterization of Repair Coating Prepared by Different Process Parameters

**Figure 3:**
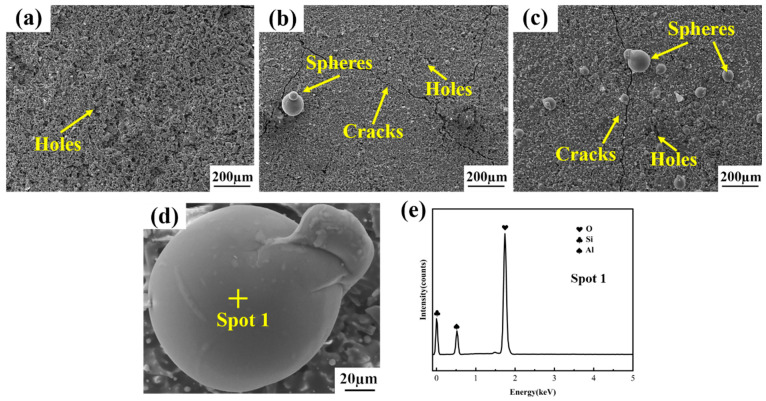
The surface morphologies of the repaired coating samples prepared by different process parameters. (**a**) Sample-1; (**b**) Sample-2; (**c**) Sample-3; (**d**) amplification diagram of spherical particles; (**e**) EDS analysis of spherical particles.

The surface morphologies of the repaired coating samples fabricated by diverse process parameters are exhibited in Figure 3. By comparing the impacts of different process parameters on the surface morphology of the repaired sample, there exist a small quantity of spherical particles and a substantial number of fissures on the surface of Sample-2 (Figure 3b). This is attributed to the relatively low temperature, which fails to reach the melting point of Si, and the non-melted Si lacks good fluidity; on the other hand, when the temperature is relatively low, the oxidation rate of Si and SiC is overly sluggish to generate a sufficient amount of the SiO_2_ glass phase in a timely manner. When the coating is subjected to thermal stress and generates microfissures, Si and SiO_2_ cannot be filled in time, and the fissures continue to propagate, resulting in a large number of fissures on the surface of the coating. With an increment in the preoxidation temperature, Sample-1 with no fissures and no spherical particles on its surface was obtained (Figure 3a), and with an increase in the Si content, a large number of spherical particles and fissures were formed on the surface of Sample-3 (Figure 3c). Upon conducting EDS analysis (Figure 3e), the spherical particles were ascertained to be Si and SiO_2_. This is because a considerable amount of silicon on the coating surface was oxidized to form the SiO_2_ glass phase on account of the high Si content. Due to the addition of Al_2_O_3_ to the original slurry, the viscosity of SiO_2_ is reduced. Si and SiO_2_ have relatively large tension during the flow process, which makes them more inclined to form spherical particles with the smallest specific surface area. Finally, a large number of Si-SiO_2_ spherical particles are formed on the surface of the coating. In comparison with the hard-to-consume SiC, Si is more difficult to continuously and effectively provide the SiO_2_ glass phase, thereby resulting in the severe cracking phenomenon of Sample-3, which provides a direct passage for the oxidation of the matrix and becomes the fundamental cause of the significant weight loss of the sample. There are small apertures on the surface of the three groups of repair coatings fabricated by different processes, which is conducive to the ingress of O_2_ into the coating, and the oxidation reaction with Si and SiC generates SiO_2_, forming a dense oxide film to safeguard the C/C substrate from oxidation.

**Figure 4:**
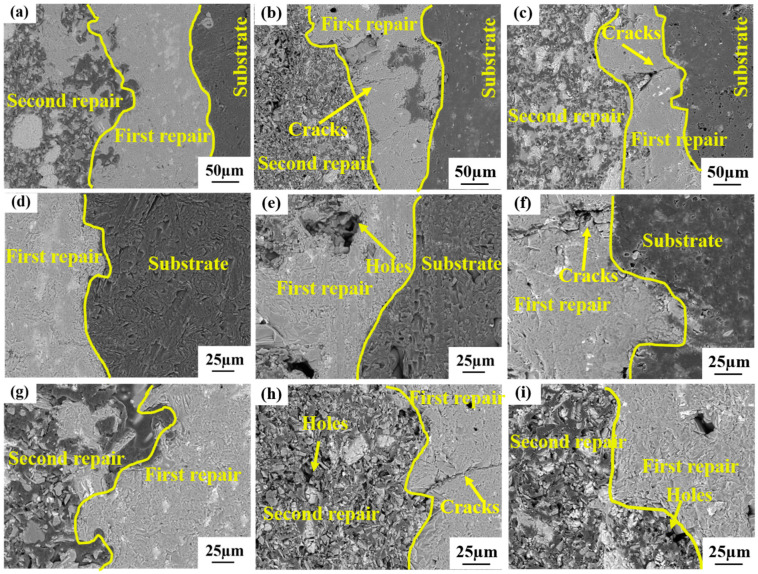
The cross-section morphologies of the repaired coating samples prepared by different process parameters: (**a**,**d**,**g**) Sample-1; (**b**,**e**,**h**) Sample-2; (**c**,**f**,**i**) Sample-3.

The cross-sectional morphologies of the repaired coating samples generated by distinct process parameters are illustrated in Figure 4. It can be noticed that there are certain small holes in the second repair coating area of Sample-1, Sample-2, and Sample-3, This is consistent with the surface morphology presented in Figure 3 and is conducive to the oxidation reaction of O_2_ with Si and SiC to form the SiO_2_ oxide film for safeguarding the C/C matrix from oxidation. Nevertheless, the adhesion between the first repair coating of Sample-1 and the C/C substrate as well as the second repair coating is extremely close (Figure 4a,d,g), and there are nearly no fissures, pores, and other flaws in the first repair coating area. The first repair coating of Sample-2 (Figure 4b,e,h) and Sample-3 (Figure 4c,f,i) is not firmly adhered to the C/C substrate and the second repair coating. Fissures, pores, and other defects emerge at the interface, and fissures, pores, and other defects also occur in the first repair coating area. These flaws will offer channels for O_2_ to come into contact with the C/C substrate through the coating, which is the fundamental cause for the severe weight loss of Sample-2 and Sample-3 in the oxidation stage, which is in correspondence with the microscopic morphology shown in Figure 3.

In contrast, a relatively lower Si content and a heightened preoxidation temperature are conducive to the genesis of a compact and sleek Si-SiC coating with fewer flaws, corresponding to Sample-1, which possesses the supreme oxidation resistance.

**Figure 5:**
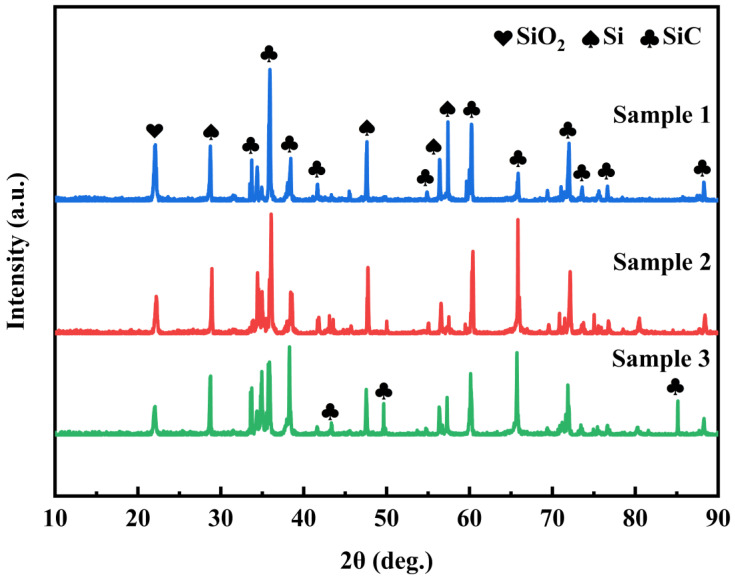
XRD patterns of repair coating samples prepared by different process parameters.

From the XRD patterns of the repaired coating samples prepared by means of different process parameters (Figure 5), it can be discerned that the composition of the three groups of samples is coincident with that of the repaired raw materials, involving SiC, Si and SiO_2_, and the predominant phase of the coating is the SiC phase. The appearance of the Si peak intimates that Si has not been fully oxidized. The SiO_2_ phase is partly in the raw material and the other part is formed via the reaction of SiC and Si. Al_2_O_3_ cannot be detected due to the relatively low content of Al_2_O_3_. The C peak was not detected in the three groups of samples, signifying that the damaged area of the sample was completely repaired and there was no exposed C/C substrate.

### 3.2. Oxidation Resistance

**Figure 6:**
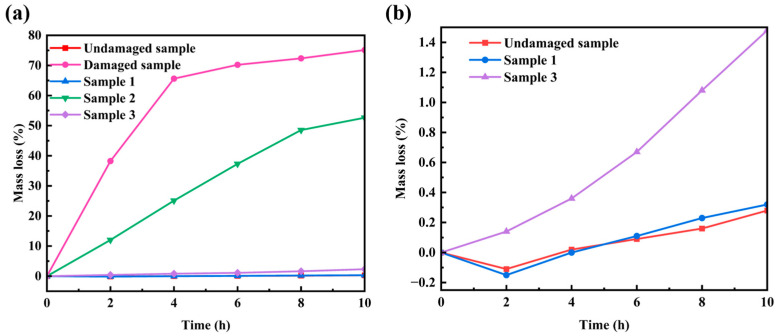
The oxidation weight loss curves of the repaired coating samples prepared by different process parameters after oxidation at 1773 K for 10 h. (**b**) is an enlarged figure of (**a**).

The oxidation weight loss curves of the damaged samples, the undamaged samples, and the three sets of repaired samples with distinct process parameters subsequent to oxidation at 1773 K for 10 h are presented in Figure 6. Figure 6b is the magnified display of Figure 6a. It can be perceived from the figure that within the temporal span of 0 to 4 h, the damaged sample undergoes severe oxidation on account of irregular cracks, pores, and other flaws in the coating’s damaged area, and a portion of the C/C substrate is exposed to the air. The weight loss is exceedingly grave, and the predominant oxidation reactions are reaction 2 and reaction 3. At this moment, the weight of both the undamaged sample and sample 1 not only fails to diminish but rather increases. This is attributed to the presence of minute pores on the surface of the repaired coating. Reaction 4 and reaction 5 transpired when O_2_ entered the coating via the small pores, which is in consonance with Figure 3. With the extension of oxidation time, the weight of the damaged sample remains essentially unaltered. This is due to the fact that as the C/C matrix is exhausted, the damaged sample almost evolves into an empty husk with merely a coating, thereby causing the subsequent oxidation weight loss of the sample to be delayed. It is observed that the damaged coating completely loses the ability of oxidation protection, and the oxidation consumption of the C/C composite matrix is substantial. The undamaged sample and Sample-1 exhibited a trifling weight loss at this stage, and the slope of the curve was minute. This is because a continuous and compact SiO_2_ film was gradually formed on the surface of the coating. On the one hand, the volatilization of Si was restrained. On the other hand, O_2_ needs to permeate through the SiO_2_ film to diffuse to the SiO_2_/Si-SiC interface in order to initiate the oxidation reaction. The small diffusion coefficient of O_2_ in SiO_2_ can effectively retard the oxidation of Si and SiC particles within the coating, and the rate of the mass loss of the sample is extremely low. Due to the mismatch of process parameters between Sample-2 and Sample-3, the coating structure in the repair area of the sample is lax and porous, not dense, and there exist a large number of holes and fissures, resulting in a considerably higher rate of weight loss than that of the dense coating sample.

(2) $$2C(s)+O_{2}\left(g\right)\to 2\mathrm{CO}(g)$$

(3) $$C(s)+O_{2}\left(g\right)\to {CO}_{2}\left(g\right)$$

(4) $$SiC(s)+{2O}_{2}\left(g\right)\to {CO}_{2}(g)+\mathrm{Si}O_{2}(s)$$

(5) $$Si(s)+O_{2}\left(g\right)\to +\mathrm{Si}O_{2}(s)$$

where “s” represents the solid state, “l” symbolizes the liquid state and “g” indicates the gaseous state.

**Figure 7:**
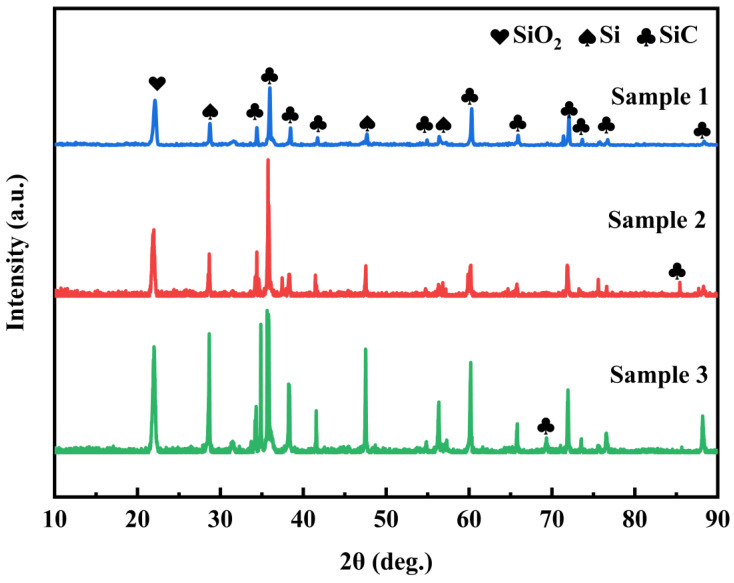
XRD patterns of repair coating samples prepared by different process parameters after oxidation at 1773 K for 10 h.

With the purpose of conducting a more in-depth analysis of the oxidation behavior of the repair coating, the microstructure of the post-oxidation repair coating was characterized. The XRD patterns of the three sets of samples are depicted in Figure 7. It can be observed from the figure that the oxidized coating is mainly composed of SiO_2_, SiC, and Si phases. The diffraction peak of SiO_2_ is partially the source of SiO_2_ in the repair coating slurry, and the other part is the product of the reaction among Si, SiC, and oxygen (reactions 4 and 5). The detection of the diffraction peaks of Si and SiC serves to prove that Si and SiC in the coating were not entirely oxidized. The Si diffraction peak of Sample-3 is significantly higher than that of Sample-1 and Sample-2. One part of the reason is that the Si content in the original slurry of Sample-3 is relatively higher, and another part is that a portion of Si in Sample-3 aggregates into spherical particles, which reduces the contact area between Si and O_2_, thereby resulting in a relatively higher remaining Si content. The diffraction peak of SiO_2_ on the surface of Sample-1 after oxidation is lower than that of Sample-2 and Sample-3. This is because a dense oxide film is formed on the surface of Sample-1 after a period of oxidation. Oxygen is unable to enter the coating through the oxide film and continue to react with Si and SiC to form SiO_2_. However, due to a large number of cracks and holes on the surfaces of Sample-2 and Sample-3, SiO_2_ cannot be filled in a timely manner, and a dense oxide film is not formed on the surface. Therefore, O_2_ can continue to react with Si and SiC to form SiO_2_, which is in line with the microstructure diagram.

**Figure 8:**
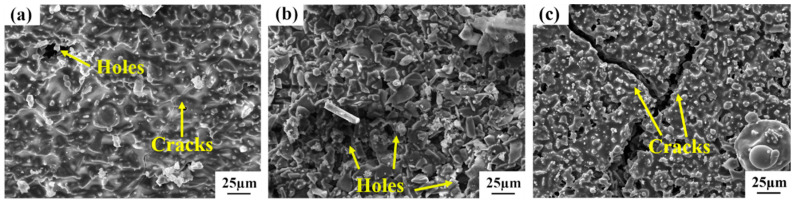
The surface morphologies of the repaired coating samples prepared by different process parameters after oxidation at 1773 K for 10 h. (**a**) Sample-1; (**b**) Sample-2; (**c**) Sample-3.

The surface morphologies of the repair coating subsequent to oxidation at 1773 K for 10 h under diverse process conditions are presented in Figure 8. Manifestly, the coating surface of each set of samples contains SiO_2_ glass film, which accords with the test outcomes of the XRD patterns. A modest amount of fissures appeared on the surface of Sample-1, which was ascribed to the fact that the thermal expansion coefficient of SiC exceeded that of the C/C matrix, engendering tensile stress on the coating when it was retrieved from the oxidation furnace and cooled, consequently giving rise to microfissures. There exist copious pores in Sample-2 and Sample-3, and the fissures are occasioned by the formation and evasion of a substantial quantity of gas-phase products such as CO, CO_2_, Si, and SiO during the oxidation process (see reactions 2, 3, 4, 6, 7, and 8). Severe oxidation corrosion leads the original pore defects in the coating structure of Sample-2 and Sample-3 to permeate with the newly generated pores on the surface, forming deep holes connected with the exterior, and an excessive number of large-volume holes are prone to becoming the initiation and propagation channels of fissures, accelerating the failure of the repair coating. In contrast, the surface pores and fissures of Sample-1 with fewer defects in the preparation process are fewer, indicating that the dense structure is conducive to inhibiting the degree of oxidation reaction and the formation of gas-phase products.

(6) $$2Si(l)+O_{2}\left(g\right)\to 2\mathrm{SiO}(g)$$

(7) $$Si(s)+{\mathrm{Si}O}_{2}\left(l\right)\to 2\mathrm{SiO}(g)$$

(8) $$SiC\left(s\right)+O_{2}\left(g\right)\to \mathrm{SiO}\left(g\right)+CO(g)$$

**Figure 9:**
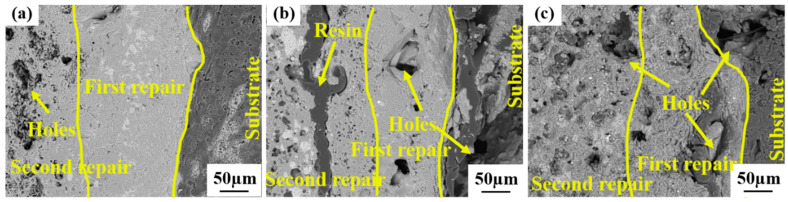
The cross-sectional morphologies of the repaired coating samples prepared by different process parameters after oxidation at 1773 K for 10 h. (**a**) Sample-1; (**b**) Sample-2; (**c**) Sample-3.

The cross-sectional morphologies of the repaired coating samples under diverse process conditions after oxidation at 1773 K for 10 h are presented in Figure 9. It can be observed from the figure that there are no large-sized defects in the first repair coating area and the C/C matrix of Sample-1; only the second repair coating area is oxidized to form holes and other defects, indicating that the repair coating of Sample-1 effectively safeguards the C/C matrix and averts its oxidation. Nevertheless, except for Sample-1, both Sample-2 and Sample-3 not only have their second repair coating area oxidized, but also the first repair coating area and the C/C matrix show massive oxidation holes, which is in correspondence with the oxidation weight loss curve of Figure 6. The large mass loss is due to the oxidation corrosion of the C/C composite material turning into a gas phase and escaping to the outside. The first repair coating area of Sample-1 is still closely bound to the C/C matrix and the second repair coating area, and there are no conspicuous defects at the interfaces. However, the first repair coating area of Sample-2 and Sample-3 is not closely adhered to the C/C substrate and the second repair coating area, and there is a significant gap between the interfaces. This is because during the preparation of the repair coating, there exist penetrating channels and cracks between the repair coating and the C/C substrate, and defects such as holes also emerge in the coating area, which directly offer a passage for O_2_ to come into contact with the C/C substrate through the coating to undergo an oxidation reaction. After a prolonged period of high-temperature oxidation, these defects are further derived and eventually lose their protective effect, thereby causing the C/C substrate to be oxidized. This reveals that reasonable process parameters can yield a dense repair coating, thereby effectively repairing the damaged sample, enhancing the oxidation resistance of the sample, and prolonging the service life of the sample.

**Figure 10:**
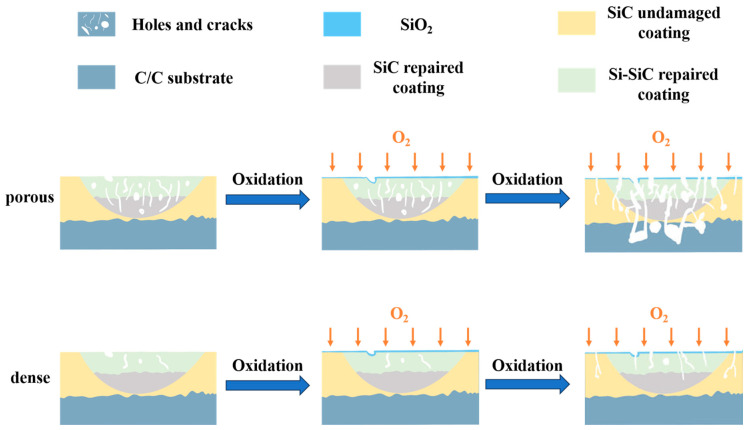
Schematic diagram of oxidation resistance mechanism of repaired samples.

A schematic diagram of the antioxidant mechanism is presented in Figure 10. The coating density is the most crucial element determining the oxidation resistance of the repair coating. That is to say, the key to enhancing the oxidation protection performance of the damaged coating is to achieve the restoration of the bare C/C composite substrate. The prerequisite for effective coverage is that the repair system has a dense structure. If there are a large number of manufacturing defects in the coating, it is extremely difficult to achieve the healing of all defects relying solely on its own limited mobile phase. The unbridged defects are prone to becoming a passage facilitating the contact between the external oxidation medium and the substrate, thereby accelerating the failure of coating repair, as depicted in Figure 10. After optimizing the process parameters, the oxidation protection ability of Sample-1 with fewer manufacturing defects is significantly enhanced and is considerably higher than that of the damaged and unrepaired sample. Therefore, if the oxidation resistance of the repaired sample is required to be the same as that of the undamaged sample, a dense microstructure must be obtained.

## 4. Conclusions

Mechanical damage in SiC-coated C/C composites was repaired through heat treatment and slurry painting–preoxidation methods. The damaged area was pretreated, and the process parameters were optimized. By adjusting the proportion of the original powder and the preoxidation temperature, a coating with a smooth surface and no obvious defects was obtained. The coating in the repaired region is closely combined with the C/C substrate and the original coating, without any obvious flaws. After oxidation in air at 1773 K for 10 h, the oxidation resistance of the repaired sample is close to that of the undamaged sample and is 74.79% lower than that of the damaged sample. Primarily, this is mainly due to the denser coating in the repaired area. This reveals that such repair technology is a reliable, convenient, and promising approach for repairing antioxidant coatings.

## Figures and Tables

**Table 1:** Slurry brushing preoxidation repair coating preparation process parameters.

Sample	Si wt/%	SiC wt/%	Al_2_O_3_ wt/%	Temperature/°C	Time/min
Sample-1	25	72	3	1450	30
Sample-2	25	72	3	1350	30
Sample-3	35	62	3	1450	30

## Data Availability

The original contributions presented in the study are included in the article, further inquiries can be directed to the corresponding author.

## References

[1] Manocha L.M. (2003). High performance carbon-carbon composites. Sadhana.

[2] Fu Y., Zhang Y., Zhang J., Chen G., Li T. (2021). Radially one-dimensional hafnium carbide-carbon/carbon networks composites for ultra-high temperature ablation-resistance. Corros. Sci..

[3] Xu Y., Sun W., Xiong X., Liu F., Luan X. (2019). Ablation characteristics of mosaic structure ZrC-SiC coatings on low-density, porous C/C composites. J. Mater. Sci. Technol..

[4] Wang H., Li H., Shi X., Liu X., Kong J., Zhou H. (2020). Repair of SiC coating on carbon/carbon composites by laser cladding technique. Ceram. Int..

[5] Hu C., Pang S., Tang S., Yang Z., Wang S., Cheng H.-M. (2015). Long-term oxidation behavior of carbon/carbon composites with a SiC/B_4_C–B_2_O_3_–SiO_2_–Al_2_O_3_ coating at low and medium temperatures. Corros. Sci..

[6] Wang H. (2023). Study on Laser Cladding Repair and Properties of MoSi_2_-SiC/SiC Coating on C/C Composites. Ph.D. Thesis.

[7] Jia Y. (2017). Investigation of Ablation Protection of Rare Earth Modified ZrC Coating System for C/C Composites. Ph.D. Thesis.

[8] Feng G., Li H., Yao X., Zhou H., Yu Y., Lu J. (2021). Ablation resistance of HfC-TaC/HfC-SiC alternate coating for SiC-coated carbon/carbon composites under cyclic ablation. J. Eur. Ceram. Soc..

[9] Xie W., Fu Q., Cheng C., Wang P., Li J., Yan N. (2021). Effect of Lu_2_O_3_ addition on the oxidation behavior of SiC-ZrB_2_ composite coating at 1500℃: Experimental and theoretical study. Corros. Sci..

[10] Ren X., Li H., Chu Y., Fu Q., Li K. (2014). Preparation of oxidation protective ZrB_2_-SiC coating by in-situ reaction method on SiC-coated carbon/carbon composites. Surf. Coat. Technol..

[11] Ren X., Li H., Fu Q., Li K. (2014). Ta_x_Hf_1−x_B_2_–SiC multiphase oxidation protective coating for SiC-coated carbon/carbon composites. Corros. Sci..

[12] Ren X., Li H., Fu Q., Li K. (2014). Oxidation protective TaB_2_–SiC gradient coating to protect SiC–Si coated carbon/carbon composites against oxidation. Compos. Part B Eng..

[13] Zhao H., Fu Z., Tang C., Liu X., Li Z., Zhang K. (2014). Study of SiC/SiO_2_ oxidation-resistant coatings on matrix graphite for HTR fuel element. Nucl. Eng. Des..

[14] Li Y., Xiao P., Li Z., Luo W., Zhou W. (2017). Oxidation behavior of C/C composites with SiC/ZrSiO_4_–SiO_2_ coating. Trans. Nonferrous Met. Soc. China.

[15] Zhang M., Ren X., Chu H., Lv J., Li W., Wang W., Yang Q., Feng P. (2020). Oxidation inhibition behaviors of the HfB_2_-SiC-TaSi_2_ coating for carbon structural materials at 1700 °C. Corros. Sci..

[16] Chen S., Qiua X., Zhang B., Xua J., Zhong F., Zhu B., Zhang Y., Ou-Yang J., Yang X. (2021). Advances in antioxidation coating materials for carbon/carbon composites. J. Alloys Compd..

[17] Jina X., Fana X., Lu C., Wang T. (2018). Advances in oxidation and ablation resistance of high and ultra-high temperature ceramics modified or coated carbon/carbon composites. J. Eur. Ceram. Soc..

[18] Monteverde F., Savino R., De Stefano Fumo M., Di Maso A. (2010). Plasma wind tunnel testing of ultra-high temperature ZrB_2_–SiC composites under hypersonic re-entry conditions. J. Eur. Ceram. Soc..

[19] Li H., Xue H., Wang Y., Fu Q., Yao D. (2007). A MoSi_2_–SiC–Si oxidation protective coating for carbon/carbon composites. Surf. Coat. Technol..

[20] Williams S.D., Curry D.M., Chao D.C., Pham V.T. (1995). Ablation analysis of the Shuttle Orbiter oxidation protected reinforced carbon-carbon. J. Thermophys. Heat Transf..

[21] Wang S., Yin J., Xiefeng M., Tang L., Xiong X., Zhang H., Wen Q., Ma D., Zuo J. (2024). Microstructure and ablation behaviour of C/C-SiC-ZrC-Cu composites prepared by reactive melt infiltration. Mater. Today Commun..

[22] Zhang P., Zhu L., Tong Y., Li Y., Xing Y., Lan H., Sun Y., Liang X. (2024). Revealing thermal shock behaviors and damage mechanism of 3D needled C/C–SiC composites based on multi-scale analysis. J. Mater. Res. Technol..

[23] Xue L., Li K., Jia Y., Zhang S., Ren J., You Z. (2016). Effects of hypervelocity impact on ablation behavior of SiC coated C/C composites. Mater. Des..

[24] Jiao X., He Q., Tan Q., Yin X. (2023). Ablation behavior of mullite modified C/C-SiC-HfC composites under oxyacetylene torch for single and cyclic ablations with two heat fluxes. J. Eur. Ceram. Soc..

[25] Wang L., Fu Q., Zhao F. (2017). A novel gradient SiC-ZrB_2_-MoSi_2_ coating for SiC coated C/C composites by supersonic plasma spraying. Surf. Coat. Technol..

[26] Lv J., Zhang Y., Li W., Zhu X., Li J., Zhang J., Li T. (2022). Microstructure evolution of HfB_2_-SiC/SiC coating for C/C composites during long-term oxidation at 1700 °C. Corros. Sci..

[27] Zhou L., Fu Q., Hu D., Wei Y., Tong M., Zhang J. (2021). Oxidation protective SiC-Si coating for carbon/carbon composites by gaseous silicon infiltration and pack cementation: A comparative investigation. J. Eur. Ceram. Soc..

[28] Choi W.J., Lee H., Park C.W., Kim Y.D., Byun J. (2021). High temperature oxidation behavior of molybdenum borides by silicon pack cementation process. Int. J. Refract. Met. Hard Mater..

[29] Zeng J., Hua J., Yang X., Xu H., Li H., Guo N., Dong Q. (2020). Microstructure and formation mechanism of the Si-Cr dual-alloyed coating prepared by pack-cementation. Surf. Coat. Technol..

[30] Xianga J., Xiea F., Wua X., Yu Y. (2019). Microstructure and tribological properties of Si-Y/Al two-step deposition coating prepared on Ti_2_AlNb based alloy by halide activated pack cementation technique. Tribol. Int..

